# Proline Metabolism in Response to Climate Extremes in Hairgrass

**DOI:** 10.3390/plants13101408

**Published:** 2024-05-18

**Authors:** Qiaoyu Luo, Yonggui Ma, Huichun Xie, Feifei Chang, Chiming Guan, Bing Yang, Yushou Ma

**Affiliations:** 1 Qinghai Provincial Key Laboratory of Medicinal Plant and Animal Resources of Qinghai-Xizang Plateau, Qinghai Normal University, Xining 810008, China; luoqycjwd@163.com (Q.L.); swxmyg@126.com (Y.M.); yezino.1@163.com (H.X.); 2017254@qhnu.edu.cn (F.C.); guancm176@163.com (C.G.); 2 School of Life Sciences, Qinghai Normal University, Xining 810008, China; 3 Academy of Plateau Science and Sustainability, Qinghai Normal University, Xining 810008, China; xinyuan890@163.com; 4 Qinghai South of Qilian Mountain Forest Ecosystem Observation and Research Station, Huzhu 810500, China; 5 College of Agriculture and Animal Husbandry, Qinghai University, Xining 810008, China; 6 Sichuan Academy of Giant Panda, Chengdu 610081, China

**Keywords:** *Deschampsia caespitosa*, proline, glutamate, ornithine, proline metabolism-related enzyme, dry–wet alternation

## Abstract

Hairgrass (*Deschampsia caespitosa*), a widely distributed grass species considered promising in the ecological restoration of degraded grassland in the Qinghai-Xizang Plateau, is likely to be subjected to frequent drought and waterlogging stress due to ongoing climate change, further aggravating the degradation of grassland in this region. However, whether it would acclimate to water stresses resulting from extreme climates remains unknown. Proline accumulation is a crucial metabolic response of plants to challenging environmental conditions. This study aims to investigate the changes in proline accumulation and key enzymes in hairgrass shoot and root tissues in response to distinct climate extremes including moderate drought, moderate waterlogging, and dry–wet variations over 28 days using a completely randomized block design. The proline accumulation, contribution of the glutamate and ornithine pathways, and key enzyme activities related to proline metabolism in shoot and root tissues were examined. The results showed that water stress led to proline accumulation in both shoot and root tissues of hairgrass, highlighting the importance of this osmoprotectant in mitigating the effects of environmental challenges. The differential accumulation of proline in shoots compared to roots suggests a strategic allocation of resources by the plant to cope with osmotic stress. Enzymatic activities related to proline metabolism, such as Δ^1^-pyrroline-5-carboxylate synthetase, ornithine aminotransferase, Δ^1^-pyrroline-5-carboxylate reductase, Δ^1^-pyrroline-5-carboxylate dehydrogenase, and proline dehydrogenase, further emphasize the dynamic regulation of proline levels in hairgrass under water stress conditions. These findings support the potential for enhancing the stress resistance of hairgrass through the genetic manipulation of proline biosynthesis and catabolism pathways.

## 1. Introduction

Global climate change is strongly associated with variations in temperature and precipitation. Climate extremes, which refer to the increasing frequency, intensity, and duration of extreme weather events [1], are expected to significantly impact both belowground and aboveground communities and processes [1,2]. For example, drought can have a negative impact on plant performance and crop yield/plant productivity [1,3]. Drought exceeding a certain threshold can reduce plant growth and alter competitive interactions when faced with continuous drought [4]. Similarly, waterlogging and flooding can have detrimental effects on growth and seed production, ultimately resulting in yield loss or even complete harvest failure [5,6,7]. Exposure of plants to waterlogging before anthesis mitigates yield loss, as some mechanisms can be primed by the first exposure [8]. Recent studies have shown that global warming has increased the temporal and spatial variability of precipitation [9,10,11]. Therefore, a comprehensive understanding of how plants respond and adapt to climate change is crucial to ensure food safety and ecosystem sustainability.

Water is one of the most critical abiotic factors that limit the survival, growth, and distribution of plants [12,13,14,15]. However, plants have a certain threshold for water demand, and both water excess and water deficit can have negative effects on plant growth, development, and production [15]. Over the long-term evolutionary process, plants have evolved a range of physiological and molecular mechanisms, such as stomatal regulation, root growth and architecture adjustments, osmotic adjustment, abscisic acid signaling, reactive oxygen species scavenging, and dormancy/germination adaptations, to effectively manage fluctuations in water availability and ensure their survival in dynamic environments. In recent decades, studies have focused on the responses of plant growth, physiology, and metabolism to water stresses [7,15,16,17,18]. However, there is a substantial gap in knowledge regarding how plants respond to a series of extreme weather events [19], such as prolonged droughts followed by sudden waterlogging due to heavy rainfalls, or waterlogging and subsequent redrying. Additionally, whether there is a sequence-dependent effect of water stress on plants is largely unknown. Finally, comparing aboveground and belowground plant components is essential for unraveling the systemic coordination of plant responses to environmental cues.

**Figure A1:**
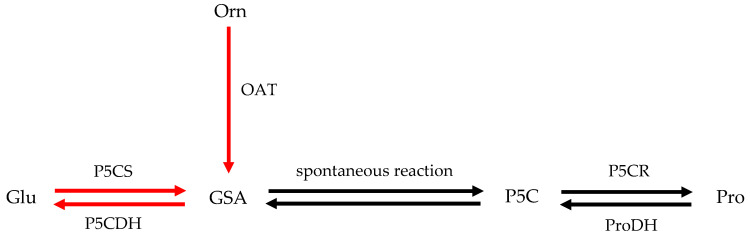
Proline metabolism.

Plants respond to osmotic and oxidative stress by synthesizing and accumulating proline (Pro), an important amino acid that assists in alleviating stress [20]. This multifunctional compound is found in high levels in plants experiencing stress [20,21,22,23,24]. Initially, Pro serves as an effective osmoprotectant, aiding plants in stress mitigation [22,25]. Moreover, Pro facilitates recovery from drought and waterlogging by serving as a source of nitrogen and carbon for growth and metabolic resumption [19,20,26]. It also plays a crucial role in maintaining cell turgor, stabilizing proteins and membranes, and scavenging reactive oxygen species during environmental stress [20,22,23,27,28,29]. Furthermore, Pro contributes to signaling pathways involved in plant stress responses [20,22,24]. For instance, the accumulation of Pro is influenced by phytohormones, calcium signaling, and mitogen-activated protein kinase pathways [20]. Finally, Pro can act as a signaling molecule, regulating gene expression and specific metabolic processes [22]. The increase in Pro content is connected to Pro biosynthesis via the glutamate (Glu) and ornithine (Orn) pathways [30,31], predominantly governed by the glutamate-based biosynthetic route [22]. Pro metabolism in plants is highly responsive to environmental fluctuations, leading to rapid changes in free Pro levels based on the prevailing conditions [22]. Typically, plants experiencing water scarcity adapt their Pro levels to alleviate osmotic stress by enhancing synthesis, reducing breakdown, and facilitating movement between various plant parts to prevent excess Pro accumulation within cells [32]. Studies have indicated that the synthesis of Pro from Glu depends on the enzymatic activities Δ^1^-pyrroline-5-carboxylate synthetase (P5CS) and Δ^1^-pyrroline-5-carboxylate reductase (P5CR), while ornithine aminotransferase (OAT) controls Pro production from Orn, initializing the Orn pathway [24,33]. In summary, Pro biosynthesis comprises two consecutive stages (Figure A1). Initially, Glu is converted to glutamic γ-semialdehyde (GSA) by the multifunctional enzyme P5CS. GSA then spontaneously forms Δ^1^-pyrroline-5-carboxylic acid (P5C), a key intermediate in Pro and Orn metabolism. Subsequently, P5C is transformed into Pro by P5CR. Alternatively, Pro can be synthesized from Orn by OAT, producing P5C, which can then be converted into Pro [20]. Pro catabolism involves two oxidative steps, sequentially catalyzed by the rate-limiting proline dehydrogenase (ProDH) and Δ^1^-pyrroline-5-carboxylate dehydrogenase (P5CDH) [22]. Studies have indicated that plants can amass Pro by inhibiting Pro breakdown/catabolism carried out by the ProDH [24,34,35].

Alpine marshes are widespread across the Qinghai-Xizang Plateau and its vicinity, a region known for its high altitudes and harsh climates. Here, the hydrological conditions of wetlands and their bordering transition areas are highly variable, often oscillating between extreme drought and sustained waterlogging [36,37]. Recent shifts toward a warmer and more humid climate on the Plateau have been attributed to global climate change [38,39]. These shifts have brought about an irregular rainfall pattern, marked by frequent droughts and a rise in intense rainfall episodes, which in turn exacerbate short-term waterlogging [40]. As climate change persists, it is anticipated to accentuate the extremes of both drought and flooding, especially in the Qinghai-Xizang Plateau, potentially exacerbating the already widespread degradation of its grasslands. Covering 60% of the Plateau, these grasslands are critical to the region, yet approximately 70% have deteriorated in recent years [41] due to the dual pressures of climate variability and human activities [42,43,44]. The consequences of grassland degradation are significant, restricting economic development, impairing herders’ livelihoods, and threatening regional stability and prosperity. Additionally, this degradation undermines the grasslands’ capacity to sustain biodiversity, ecosystem services, and human well-being [45]. In response to these issues, since 2004, the Chinese National Forestry and Grassland Administration has initiated a range of ecological restoration strategies, including improved grassland management and fencing. However, challenges remain in selecting appropriate plant species and designing effective landscapes to combat ecosystem degradation. Consequently, these challenges have become focal points in Chinese ecological research and policy development [42]. Hairgrass (*Deschampsia caespitosa*), with its notable cold and trimming resistance, is regarded as a viable candidate for the ecological restoration of these degraded grasslands in the Qinghai-Xizang Plateau. Despite its potential, the adaptability of hairgrass to the water stresses induced by extreme climatic conditions has yet to be determined.

The primary objective of the present study is to investigate the changes in Pro accumulation and associated key enzymes in the leaves and roots of hairgrass in response to distinct water stresses. The specific questions addressed are (1) whether water stress would induce Pro accumulation and increase associated substance contents and key enzymatic activities in hairgrass compared to well-irrigated conditions, (2) whether dry–wet transition induces Pro accumulation and increases associated substance contents and key enzymatic activities in hairgrass compared to lasting drought or waterlogging stress, and (3) whether there is any difference in the response of Pro metabolism between aboveground and underground parts.

## 2. Materials and Methods

### 2.1. Plant Materials and Growth Conditions

The experimental plant utilized in the present study is hairgrass, a novel species developed through natural growth and human cultivation. The Grassland Institute, part of the Academy of Veterinary Sciences at Qinghai University, generously provided the seeds for this study. Seeds with strong seed vitality, high germination rates, and no signs of disease were carefully selected for further experimentation. The seeds were surface sterilized by immersing them in a 2% aqueous solution of sodium hypochlorite for 10 min, followed by rinsing with sterile de-ionized water five times for 5 min each. The soil mixture used in this study consisted of equal parts sand and loam sourced from an alpine meadow in Dawu town, Maqin County, Qinghai Province. The sand and soil were thoroughly mixed until a homogeneous mix was achieved, and then placed in pots. The mix exhibited specific physicochemical characteristics, including total nitrogen at 3.12 mg/g, total phosphorus at 0.26 mg/g, total potassium at 19.58 mg/g, organic matter at 14.53 mg/g, a pH level of 7.63 (measured with a water–soil ratio of 1:1 volume/weight), and a CEC of 225.52 μS/cm (measured with a water–soil ratio of 5:1 volume/weight).

### 2.2. Experimental Details

The experimental site, situated at Qinghai Normal University (36°44′31.2″ N, 101°44′56.4″ E), is at an altitude of 2390 m above sea level, experiencing an average summer temperature of 16.4 °C. This study utilized a completely randomized block design with 10 replicates per treatment. Four water stress scenarios (Table 1), namely moderate waterlogging (MW), moderate drought (MD), alternating moderate waterlogging and drought (MW-MD), and alternating moderate drought and waterlogging (MD-MW) were set up. Non-stressed plants (without waterlogging and drought) served as the control group. In September 2018, hairgrass seeds were directly sown in pots with perforated bottoms (20 mm diameter, 25 mm height) with 3.0 kg of growth medium. Germination occurred within 3–5 days. Once the seedlings were established, they were thinned to 10 plants per pot and maintained with consistent watering. The plants were overwintered in a greenhouse and returned outdoors in mid-April 2019. The watering treatments commenced on 25 July 2019, when the hairgrass reached 25 cm in height, and continued for 28 days. To simulate drought and waterlogging conditions, a tray or bucket was placed inside the plastic basin, respectively, to manage water levels. Throughout the watering process, a canopy was constructed for ventilation without impacting temperature and humidity, and real-time weather data were monitored using a portable weather meter (holder HED-SQ, Shandong Holder Electronic Technology Co., Ltd, Weifang, China). Soil water content was measured with a soil water sensor (Pro Check, Decagon Devices, Inc., Washington, USA) to adjust water levels accordingly. A bare soil surface in the basin served as an evaporation control, with watering taking place from 18:00 to 19:00. Plant morphological, growth, and physiological characteristics were monitored and recorded during watering sessions. On the 29th day, marking the maximum duration of stress exposure, the plants were harvested and rinsed, first with tap water and then with distilled water, and surface moisture was removed. The samples were separated into shoots and roots, flash-frozen in liquid nitrogen, and stored at −80 °C for subsequent analysis. Any excess moisture on the surface was then dried. Each sample’s shoots and roots were placed into separate cryopreservation tubes, rapidly frozen in liquid nitrogen, and subsequently stored in a refrigerator at −80 °C for future steps.

### 2.3. Content Determination of Pro, Glu, Orn, GSA, and P5C

Measuring compounds such as Pro, Glu, Orn, GSA, and P5C in hairgrass under water stress conditions offers valuable insights into the physiological and biochemical responses of the plant to water scarcity or waterlogging. Specifically, Pro acts as a well-known osmoprotectant that accumulates in plants under stress conditions, including water stress, to maintain cell turgor and protect cellular structures from dehydration-induced damage. Glu serves as a precursor for proline biosynthesis and plays a role in various metabolic processes in response to stress. Changes in Glu levels can indicate shifts in nitrogen metabolism and amino acid balance under water stress. Orn, a key intermediate in the Pro biosynthesis pathway via the Orn pathway, provides insights into the regulation of Pro accumulation and stress responses in plants. GSA, an important intermediate in the Pro biosynthesis pathway, reflects the activity of enzymes involved in Pro synthesis and serves as a marker for stress-induced metabolic changes. P5C, another intermediate in Pro biosynthesis, is closely connected to the regulation of Pro levels in response to environmental stresses like water scarcity. Monitoring P5C levels assists in understanding the dynamics of Pro metabolism under water stress conditions. In essence, analyzing these specific compounds in hairgrass subjected to water stress enables researchers to gain a thorough understanding of the plant’s biochemical and physiological responses. This information facilitates the study of stress tolerance mechanisms, the identification of potential biomarkers for drought resistance, and the development of strategies to enhance plant resilience to water scarcity.

The Pro content was extracted from 0.5 g fresh tissues in 3% (*w*/*v*) aq. sulfosalicylic acid and estimated by using a ninhydrin reagent according to the protocol as described elsewhere [46]. The absorbance of the fraction with toluene aspired from the liquid phase was read at 515 nm. Proline concentration was determined using a calibration curve. The contents of Glu, Orn, GSA, and P5C were determined following the protocol and instructions of an enzyme-linked immunoassay kit produced by Shanghai Jianglai Biotech Co., Ltd., Shanghai, China.

### 2.4. Assay of Pro Metabolism-Related Enzymatic Activities

The enzyme solution was prepared following the protocol as described elsewhere [47], where 0.5 g of tissue was homogenized in 10 mL of 0.1 M KH_2_PO_4_ buffer (pH = 7.8) on ice. After centrifugation at 4000 rpm for 15 min, Triton X-100 was added to the supernatant to a final concentration of 0.15%. The resulting mixture was incubated in an ice bath for 30 min, then further centrifuged at 20,000 rpm for 20 min to obtain the supernatant used for enzyme activity determination. The activities of P5CDH, P5CR, and P5CS were determined using an enzyme-linked immunoassay kit and the method in [48], respectively. OAT activity was assessed following the procedure outlined in [49], where the reaction was initiated by adding a Tris-KOH buffer (pH = 8.0) containing ornithine, α-ketoglutarate, and NADPH I to the crude enzyme solution. The OAT activity was monitored by measuring changes in absorbance at 340 nm within 1 min. Furthermore, the activity of proDH was determined [47], with the reaction initiated by adding a Na₂CO₃ buffer (pH = 10.3), proline, and NAD^+^ to the crude enzyme solution. The activity of proDH was determined by monitoring changes in absorbance at 340 nm over a 1 min period.

### 2.5. Statistical Analysis

The effects of water treatment on variables of plants were examined with a one-way ANOVA or Kruskal–Wallis H-test. The assumptions of the ANOVA were checked using a Shapiro–Wilk test and Levene’s test before analysis. Differences between water treatments were further examined according to Fisher’s Least Significant Difference (LSD) tests at *p* < 0.05. Shoots and roots were compared by an independent sample *t*-test or Mann–Whitney U-test. All the statistical analyses were performed with the software package SPSS 22.0 (IBM, Armonk, NY, USA). The figures were prepared using OriginPro 2017 (OriginLab Corp., Northampton, MA, USA). Additionally, Spearman correlation coefficients between variables characterizing the Pro metabolism of plants were examined in R 4.1.3 (R Core Team, 2020, Alcatel-Lucent Bell Labs, Murray Hill, NJ, USA).

## 3. Results

### 3.1. Changes in Pro Content

**Figure 1:**
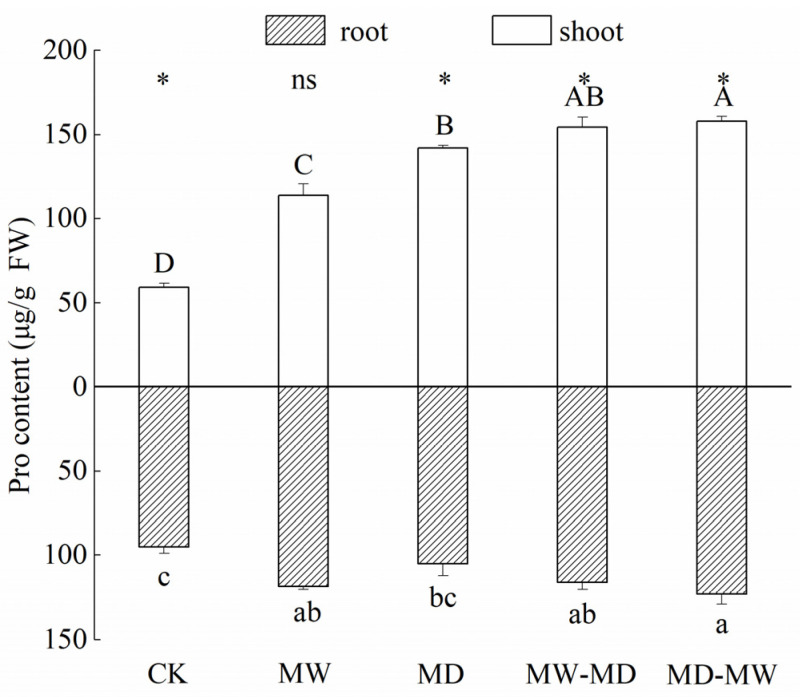
Proline (Pro) content (mean ± standard error (SE), *N* = 3) of hairgrass (*D. caespitosa*) under distinct climate extreme events. CK, control; MW, medium waterlogging stress; MD, moderate drought stress; MW-MD, alternating moderate waterlogging and drought stress; MD-MW, alternating moderate drought and waterlogging stress. Different uppercase letters indicate that there are significant differences in the shoots of hairgrass between treatments (*p* < 0.05), different lowercase letters indicate that there are significant differences in the roots of hairgrass between treatments (*p* < 0.05). * indicates that there are significant differences between shoots and roots (*p* < 0.05), and ns indicates that there are no significant differences between shoots and roots (*p* ≥ 0.05).

Pro contents in both the shoots and roots of hairgrass were responsive to water stress (Table 2; Figure 1). Overall, the Pro content in both the shoots and roots of hairgrass increased significantly under water stress, and the increase in the Pro content in the root system was more notable. Additionally, the increase in Pro content in the shoots of hairgrass under MW-MD and MD-MW conditions was more remarkable than that under MW and MD stress, respectively, whereas a significant difference in the Pro content in the roots of hairgrass was only observed between MD-MW and MD. Furthermore, there were significant differences in Pro content in shoots and roots under the same condition except for lasting waterlogging stress. Finally, the proportional increase in Pro content in the shoots was greater in comparison with that in the roots.

### 3.2. Changes in Contents of Pro Metabolic Substrates and Intermediate Product

**Figure 2:**
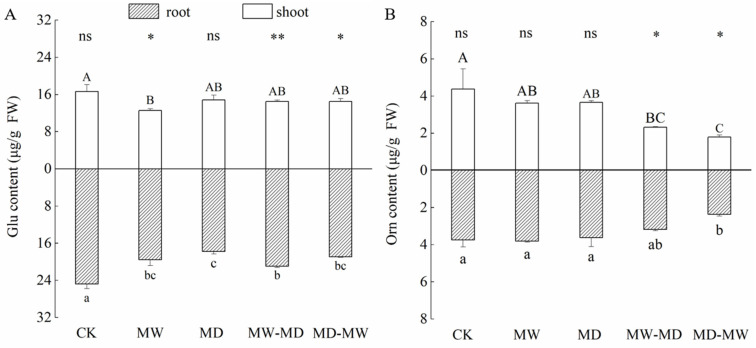
Contents (mean ± standard error (SE), *N* = 3) of glutamate (Glu, (**A**)) and ornithine (Orn, (**B**)) in shoots and roots of hairgrass (*D. caespitosa*) under distinct climate extreme events. CK, control; MW, medium waterlogging stress; MD, moderate drought stress; MW-MD, alternating moderate waterlogging and drought stress; MD-MW, alternating moderate drought and waterlogging stress. Different uppercase letters indicate that there are significant differences in the shoots of hairgrass between treatments (*p* < 0.05), different lowercase letters indicate that there are significant differences in the roots of hairgrass between treatments (*p* < 0.05). ** indicates that there are extremely significant differences between shoots and roots (*p* < 0.01), * indicates that there are significant differences between shoots and roots (*p* < 0.05), and ns indicates that there are no significant differences between shoots and roots (*p* ≥ 0.05).

Water stress affected the contents of Glu and Orn in the shoots and roots differently (Table 2; Figure 2). Overall, lasting drought induced no significant decline in Glu content in shoots of hairgrass, whereas both drought and waterlogging significantly suppressed Glu contents in roots of hairgrass. Additionally, the Glu contents in shoots were lower than that in roots under the same condition except for the CK and MD. In contrast, a significant difference in Orn contents between shoot and root was observed only under MW-MD stress.

During dry–wet cycling, both MW-MD stress and MD-MW stress markedly impacted the contents of Orn in the shoots of hairgrass, whereas only moderate drying and sequential rewetting stress (MD-MW) reduced the contents of Orn in the roots of hairgrass (Figure 2).

**Figure 3:**
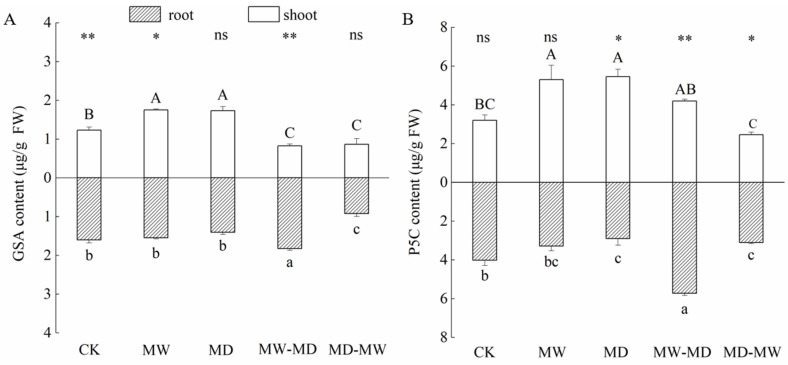
Contents (mean ± standard error (SE), *N* = 3) of glutamic-γ-semialdehyde (GSA, (**A**)) and Δ^1^-pyrroline-5-carboxylic acid (P5C, (**B**)) in shoots and roots of hairgrass (*D. caespitosa*) under distinct climate extreme events. CK, control; MW, medium waterlogging stress; MD, moderate drought stress; MW-MD, alternating moderate waterlogging and drought stress; MD-MW, alternating moderate drought and waterlogging stress. Different uppercase letters indicate that there are significant differences in the shoots of hairgrass between treatments (*p* < 0.05), different lowercase letters indicate that there are significant differences in the roots of hairgrass between treatments (*p* < 0.05). ** indicates that there are highly significant differences between shoots and roots (*p* < 0.01), * indicates that there are significant differences between shoots and roots (*p* < 0.05), and ns indicates that there are no significant differences between shoots and roots (*p* ≥ 0.05).

Under lasting moderate waterlogging stress and lasting moderate drought stress, the contents of both GSA and P5C in the shoots were significantly higher than those in the shoots of CK, whereas under dry–wet cycling, the GSA content and the P5C content in the shoots responded distinctly (Figure 3). MD and MW significantly increased the contents of GSA and P5C in shoots of hairgrass compared to CK. By contrast, only MD significantly decreased the P5C content in roots of hairgrass. In shoots of hairgrass, both MW-MD stress and MD-MW stress markedly reduced GSA content (Figure 3A), while only MD-MW stress triggered reduced P5C contents (Figure 3B). The MD-MW stress significantly decreased the GSA content in the roots of hairgrass, while lasting moderate drought significantly decreased the GSA content in the roots of hairgrass (Figure 3A). Interestingly, the MW-MD triggered a noticeable overproduction for both GSA and P5C in roots.

### 3.3. Changes in Activities of Pro Metabolism-Related Enzymes

**Figure 4:**
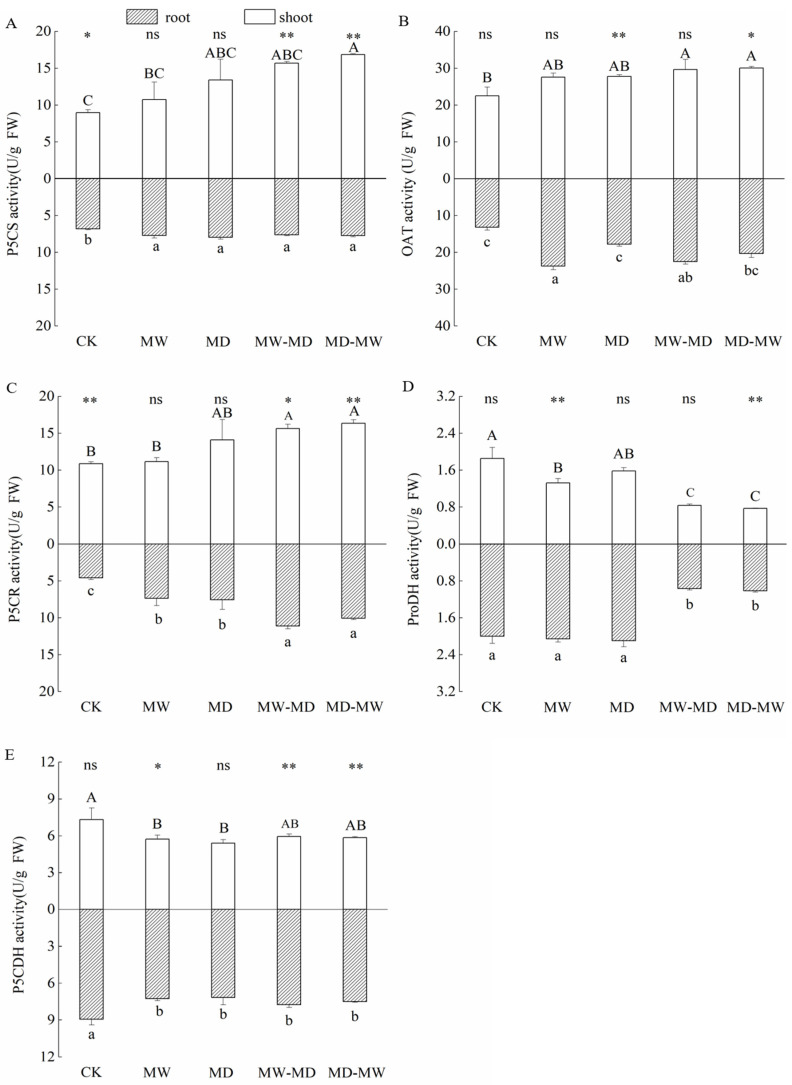
Enzymatic activity (mean ± standard error (SE), *N* = 3) of Δ^1^-pyrroline-5-carboxylate synthetase (P5CS, (**A**)), ornithine aminotransferase (OAT, (**B**)), Δ^1^-pyrroline-5-carboxylate reductase (P5CR, (**C**)), proline dehydrogenase (ProDH, (**D**)), and Δ^1^-pyrroline-5-carboxylate dehydrogenase (P5CDH, (**E**)) in shoots and roots of hairgrass (*D. caespitosa*) under distinct climate extreme events. CK, control; MW, medium waterlogging stress; MD, moderate drought stress; MW-MD, alternating moderate waterlogging and drought stress; MD-MW, alternating moderate drought and waterlogging stress. Different uppercase letters indicate that there are significant differences in the shoots of hairgrass between treatments (*p* < 0.05), different lowercase letters indicate that there are significant differences in the roots of hairgrass between treatments (*p* < 0.05). ** indicates that there are highly significant differences between shoots and roots (*p* < 0.01), * indicates that there are significant differences between shoots and roots (*p* < 0.05), and ns indicates that there are no significant differences between shoots and roots (*p* ≥ 0.05).

The response of Pro metabolism-related enzymes was dependent on water stress (Table 3). The MD-MW stress significantly increased the activities of P5CS (Figure 4A), OAT (Figure 4B), and P5CR (Figure 4C) but significantly inhibited that of ProDH (Figure 4D) in the shoots of hairgrass. MW-MD stress significantly activated the activities of OAT (Figure 4B) and P5CR (Figure 4C) but inhibited that of ProDH (Figure 4D) in the shoots of hairgrass. The ProDH (Figure 4D) activity under lasting moderate waterlogging stress was significantly inhibited in comparison with CK, whereas it was stimulated in comparison with that under MD-MW stress and MW-MD stress. The activity of P5CDH in shoots of hairgrass was significantly inhibited by MW in comparison with CK (Figure 4E). Additionally, MD significantly inhibited the activity of P5CDH in shoots of hairgrass (Figure 4E). Water stress significantly stimulated the activity of P5CS (Figure 4A) and P5CR (Figure 4C), whereas it significantly inhibited the activity of P5CDH in roots of hairgrass (Figure 4E). MW-MD stress and MD-MW stress significantly stimulated P5CR (Figure 4C) in comparison with MW and MD.

Additionally, the response of Pro metabolism-related enzymes was significantly different between shoots and roots and across water stresses (Table 3; Figure 4). There were significant differences in P5CS activity between shoots and roots under CK, MW-MD, and MD-MW (Figure 4A). There were significant differences in OAT activity (Figure 4B) between shoots and roots under CK, lasting moderate drought, and MD-MW. There were significant differences in P5CR activity (Figure 4C) between shoots and roots under CK, lasting moderate waterlogging, MW-MD, and MD-MW. There were significant differences in ProDH (Figure 4D) between shoots and roots under water stress, including lasting moderate waterlogging, lasting moderate drought, MW-MD, and MD-MW. There were significant differences in P5CDH (Figure 4D) between shoots and roots under lasting moderate waterlogging, MW-MD, and MD-MW.

### 3.4. Correlation between Metabolites and Key Enzymes in Pro Metabolism

**Figure 5:**
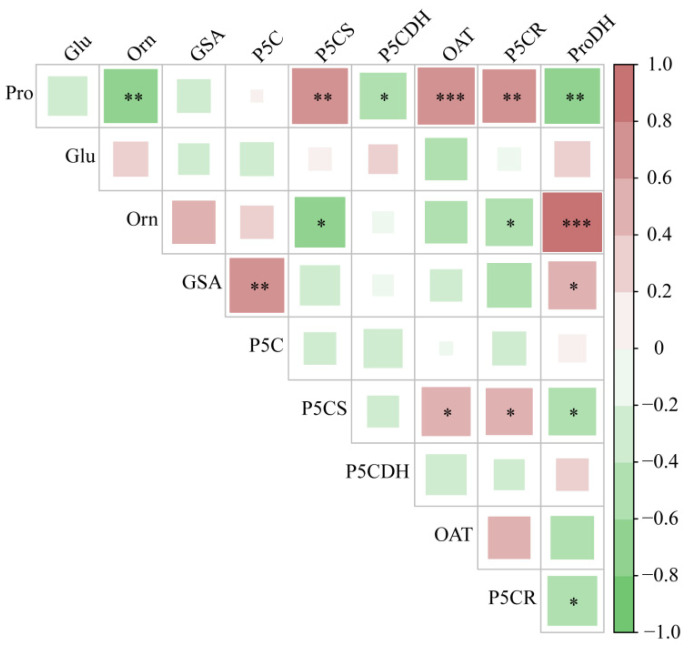
Correlation analysis of metabolites and key enzymes in the Pro metabolic pathways in shoots of hairgrass (*D. caespitosa*). Pro, proline; Glu, glutamate; Orn, ornithine; GSA, glutamic γ-semialdehyde; P5C, Δ^1^-pyrroline-5-carboxylic acid; P5CS, Δ^1^-pyrroline-5-carboxylate synthetase; P5CDH, Δ^1^-pyrroline-5-carboxylate dehydrogenase; OAT, ornithine aminotransferase; P5CR, Δ^1^-pyrroline-5-carboxylate reductase; ProDH, proline dehydrogenase. *** indicates that there are extremely significant differences between shoots and roots (*p* < 0.001), ** indicates that there are extremely significant differences between shoots and roots (*p* < 0.01), * indicates that there are significant differences between shoots and roots (*p* < 0.05).

As shown in Figure 5, the Pro content in the shoots of hairgrass was significantly positively correlated with the activities of P5CS and P5CR (*p* < 0.01). The Orn content in the shoots of hairgrass was negatively correlated with ProDH activity *(p* < 0.01) and P5CDH activity (*p* < 0.05). The GSA content was significantly positively correlated with the P5C content (*p* < 0.01), and the ProDH activity was significantly positively correlated with the GSA content (*p* < 0.05). The activity of P5CS was positively correlated with the activity of OAT and P5CR (*p* < 0.05) and negatively correlated with the activity of ProDH (*p* < 0.05). P5CR activity was negatively correlated with ProDH activity (*p* < 0.05).

**Figure 6:**
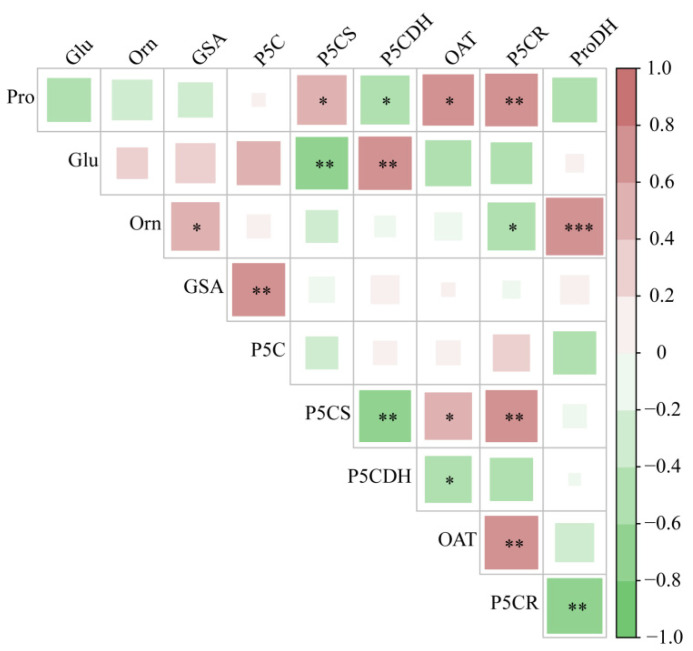
Correlation analysis of metabolites and key enzymes in the Pro metabolic pathways in roots of hairgrass (*D. caespitosa*). Pro, proline; Glu, glutamate; Orn, ornithine; GSA, glutamic γ-semialdehyde; P5C, Δ^1^-pyrroline-5-carboxylic acid; P5CS, Δ^1^-pyrroline-5-carboxylate synthetase; P5CDH, Δ^1^-pyrroline-5-carboxylate dehydrogenase; OAT, ornithine aminotransferase; P5CR, Δ^1^-pyrroline-5-carboxylate reductase; ProDH, proline dehydrogenase. *** indicates that there are extremely significant differences between shoots and roots (*p* < 0.001), ** indicates that there are extremely significant differences between shoots and roots (*p* < 0.01), * indicates that there are significant differences between shoots and roots (*p* < 0.05).

As presented in Figure 6, there was a significant negative correlation between Pro content and P5CR activity in the roots of hairgrass (*p* < 0.01), a significant positive correlation with P5CS and OAT activity (*p* < 0.05), and a significant negative correlation with P5CDH activity (*p* < 0.05). The Glu content was positively correlated with P5CDH activity (*p* < 0.01) and negatively correlated with P5CS activity (*p* < 0.01). The Orn content was positively correlated with ProDH activity (*p* < 0.01) and negatively correlated with P5CR activity (*p* < 0.05). P5CS activity was significantly positively correlated with P5CR activity (*p* < 0.01) and OAT activity (*p* < 0.05), whereas it was significantly negatively correlated with ProDH activity (*p* < 0.01). P5CDH activity was negatively correlated with OAT activity (*p* < 0.05). The OAT activity was negatively correlated with P5CR activity (*p* < 0.05). P5CR activity was negatively correlated with ProDH activity (*p* < 0.01).

## 4. Discussion

In the current investigation, the responses of Pro substrates and their immediate derivatives under diverse irrigation regimes were examined. By elucidating the complex processes that govern Pro metabolism in plants facing variable environmental stressors, this study endeavors to shed light on the mechanisms of plant adaptation and stress resilience. The findings hold potential implications for enhancing crop yields and bolstering ecosystem robustness.

### 4.1. Proline Accumulation

The accumulation of proline contributes to the stress tolerance of plants [20,22]. The results of this study indicate that water stress led to significant changes in proline content within the shoot and root systems. Overall, the increase in proline content in the shoots was more pronounced when compared to the roots (Figure 1). These findings align with the existing literature suggesting that proline accumulation is a common response to water stress [50,51,52,53,54,55,56,57,58,59]. This observed preferential increase in proline in the shoots of plants under water stress, compared to the roots, mirrors similar trends seen in *Plantago fengdouensis* [60] and wheat [56,61], suggesting that shoot growth in hairgrass exhibits a superior sensitivity to proline accumulation under water stress conditions. This sensitivity may be considered a survival strategy employed by plants. Additionally, the findings of this study also reveal that proline accumulation is particularly prominent when plants experience moderate waterlogging followed by moderate drought stress as well as moderate drying and subsequent rewetting stress (Figure 1). Previous research has shown that waterlogging post-anthesis can lead to a decrease in plant growth and the redistribution of dry matter [62]. In a recent study [19], it was found that drought followed by waterlogging led to increased proline levels in the leaves of waterlogged tomato plants. Contrary to the suggestion that the effects of these stresses would balance out when faced with the opposite stress, our findings, along with a previous study, suggest that plants exposed consecutively to two opposing stresses struggle to recover to normal growth levels [19]. This could be due to the initial stress impeding growth, physiology, and metabolism. Both rewetting after drought and redrying after waterlogging can act as additional stressors.

### 4.2. Changes in Metabolite-Related Substrate Contents and Enzymatic Activities

The accumulation of proline under water stress is associated with proline biosynthesis and the rate of oxidative degradation [63]. Previous research has indicated that proline can be accumulated through de novo synthesis, reduced degradation, or a combination of both [20,27]. Additionally, studies have shown that both increased proline biosynthesis and decreased proline catabolism occur under drought conditions [64]. Furthermore, the biosynthesis of proline and polyamines is interconnected, with proline degradation during stress promoting polyamine production [65].

P5CS has been identified as crucial for Pro synthesis [66], and enhancing the activities of P5CS, P5CR, and OAT synthase while reducing ProDH levels can lead to higher Pro biosynthesis [24]. In this study, we observed significant increases in the activities of the rate-limiting enzyme P5CS in the Glu pathway, the key enzyme OAT in the Orn pathway, and P5CR in the Pro pathway. Conversely, the activities of ProDH and P5CDH in proline catabolism were significantly reduced (Figure 4). Additionally, we found a positive correlation between Pro content in hairgrass shoots and the activities of P5CS and P5CR, while Orn content was negatively correlated with ProDH and P5CDH activities (Figure 5). These findings suggest that Pro accumulation results from enhanced proline biosynthesis and decreased oxidative degradation processes. The findings of this study are consistent with [67], suggesting that water stress induces Pro accumulation due to combined effects of increased Pro biosynthesis and its decreased degradation. It was observed in the present study that the synthesis of Pro relied on the upregulation of both P5CS and P5CR. A separate study [55] also showed a positive relationship between Pro levels and P5CS activity in the roots and leaves of plants. Conversely, Pro breakdown was attributed to the reverse activity of ProDH and P5CDH [68]. In response to drought stress, the upregulation of the gene encoding P5CS and the downregulation of the gene encoding ProDH were observed in the leaves of two drought-tolerant cowpea cultivars [69]. Drought stress led to increased activities of OAT and P5CS but inhibited ProDH activity in creeping bentgrass [59]. It is widely accepted that the expression of P5CS mRNA and OAT mRNA in plants may be influenced by nitrogen levels, with the Glu pathway being the primary pathway under osmotic stress and low-nitrogen conditions, while the Orn pathway prevails under impermeable stress and high-nitrogen conditions [70].

Pro is an amino acid that plays a pivotal role in plant stress responses, serving as an osmoprotectant or signaling molecule. Orn, another amino acid, is involved in the urea cycle and Pro synthesis within plant systems. Glu is a crucial amino acid for Pro synthesis. GSA is an intermediate in Pro biosynthesis derived from Glu, while P5C is a central intermediate in both the synthesis and catabolism of Pro. P5CS catalyzes the rate-limiting step in Pro biosynthesis from Glu. P5CR then reduces P5C to Pro, while ProDH mediates the oxidation of Pro back to P5C, initiating its catabolism. P5CDH facilitates the second step in Pro degradation, converting P5C back to Glu, and OAT is implicated in Pro biosynthesis from Orn. In the shoots, the observed positive correlation between proline content and the activities of P5CS and P5CR suggests that increased Pro synthesis may be attributed to the upregulation of these enzymes. Conversely, the negative correlation between Orn content and the activities of ProDH and P5CDH implies that active Pro degradation (mediated by ProDH and P5CDH) leads to decreased levels of Orn, a Pro precursor, in the shoots. The positive correlation between GSA and P5C contents indicates a direct relationship in the Pro synthesis pathway, as GSA is converted to P5C. Furthermore, the positive correlation of ProDH activity with GSA content suggests that an increase in Pro degradation may coincide with elevated GSA levels, potentially due to feedback mechanisms. In the roots, the negative correlation between Pro content and P5CR activity is noteworthy, highlighting a potentially complex regulation of Pro levels that may vary between different plant organs, suggesting organ-specific regulatory mechanisms. The positive correlation between Pro content and the activities of P5CS and OAT in the roots indicates active Pro synthesis in this part of the plant. In contrast, the negative correlation between Pro content and P5CDH activity supports the role of P5CDH in Pro degradation, which contributes to reduced Pro levels. The positive correlation of Glu content with P5CDH activity suggests that Glu production accompanies Pro degradation. The positive correlation between Orn content and ProDH activity, along with the negative correlation with P5CR activity, suggests a dynamic equilibrium between Pro synthesis and degradation, with Orn levels increasing as Pro is broken down. Collectively, these results indicate a tightly regulated Pro metabolism in hairgrass, characterized by significant interactions between biosynthetic and catabolic pathways and their respective enzymes. This regulation is likely critical for the plant’s response to various stressors, considering Pro’s role in stress resilience. The data suggest that these metabolic processes are finely adjusted and may exhibit differential regulation in the shoots compared to the roots. These insights are essential for understanding how hairgrass adapts to environmental stress and could inform agricultural practices related to stress tolerance mechanisms in other crops. In conclusion, this study on Pro metabolism in hairgrass reveals a sophisticated and tightly regulated system involving multiple enzymes and metabolic pathways. The interactions between Pro synthesis and degradation pathways exhibit organ-specific regulation, with implications for the plant’s evolved mechanisms to modulate Pro levels in response to stress, where feedback mechanisms likely have a role. Understanding the detailed regulation of Pro metabolism in hairgrass could offer valuable insights for enhancing stress tolerance in other crops through targeted genetic or agronomic strategies. Further research into the specific regulatory mechanisms governing Pro metabolism in hairgrass could elucidate how plants manage environmental stresses and contribute to the development of more resilient crop varieties in the future.

### 4.3. Merits and Limitations

The biggest merit of the present study is that we examine whether and how plants can cope with different subsequent stresses, including drought and rewetting and waterlogging and redrying. The effects of drought followed by waterlogging have rarely been studied yet [19]. Unfortunately, the findings of the present study may not truly reveal how plants adjust to challenging environments and withstand external threats because we did not consider the realistic spatiotemporal characteristics of drought and waterlogging events under the background of climate change. Further study with an intricate design is also needed. Despite this limitation, the findings of the present study provide some very important insights which can be used to breed water-stress-tolerant plants.

## 5. Conclusions

Simulated water stress resulted in Pro accumulation in the shoots and roots of hairgrass, as it promoted proline synthesis and inhibited its degradation. Shoots exhibited significantly higher Pro levels than roots under the same water stress, indicating a survival strategy for plants under osmotic stress. Pro metabolism showed a heightened response to MW, followed by MD, and subsequent MD-MW. Changes in enzymatic activities related to Pro metabolism explained the contributions of Pro biosynthesis through the Glu and Orn pathways, as well as catabolism. Understanding how plants respond to various water conditions due to climate change can aid in the development of tolerant plant varieties through breeding efforts.

## Figures and Tables

**Table 1:** Experimental set-up.

Treatment	Watering Schedule
Control, CK	The irrigation threshold was designed at 70–80% field capacity during the whole period of the experiment
Moderate waterlogging, MW	The irrigation threshold was designed at 80% field capacity during the whole period of the experiment and subjected to waterlogging after anthesis (maintenance of an approximately 3 cm water level below the soil surface)
Moderate drought, MD	Plants were irrigated at 50% of the control, that is, at 30–40% field water capacity for the whole period of the experiment
Alternating moderate waterlogging and drought, MW-MD	Plants were exposed to waterlogging for 14 days, and then they were subjected to drought for 14 days
Alternating moderate drought and waterlogging, MD-MW	Plants were exposed to drought for 14 days, and then they were subjected to waterlogging for 14 days

**Table 2:** Summary of one-way ANOVAs examining the effects of drought/waterlogging on the contents of proline (Pro), glutamate (Glu), ornithine (Orn), glutamic-γ-semialdehyde (GSA), and Δ^1^-pyrroline-5-carboxylic acid (P5C) in shoot and root tissues of hairgrass (*D. caespitosa*).

Variables	Shoot			Root		
	*df*	*F*	*p*	*df*	*F*	*p*
Pro	4	86.89	<0.001	4	5.322	0.015
Glu	4	2.697	0.093	4	13.104	0.001
Orn	4	4.591	0.023	4	4.594	0.023
GSA	4	23.364	<0.001	4	31.855	<0.001
P5C	4	10.501	0.001	4	23.877	<0.001

**Table 3:** Summary of one-way ANOVAs examining the effects of drought/waterlogging on activities of Δ^1^-pyrroline-5-carboxylate synthetase (P5CS), Δ^1^-pyrroline-5-carboxylate dehydrogenase (P5CDH), ornithine aminotransferase (OAT), Δ^1^-pyrroline-5-carboxylate reductase (P5CR), and proline dehydrogenase (ProDH) in shoot and root tissues of hairgrass (*D. caespitosa*).

Variables	Shoot			Root		
	*df*	*F*	*p*	*df*	*F*	*p*
P5CS	4	3.959	0.035	4	4.325	0.027
P5CDH	4	2.478	0.111	4	4.054	0.033
OAT	4	3.079	0.068	4	25.420	<0.001
P5CR	4	3.706	0.042	4	11.047	0.001
ProDH	4	14.706	<0.001	4	37.440	<0.001

## Data Availability

Data are contained within the article.
